# Supplementary material for: Defective excitation-contraction coupling and mitochondrial respiration precede mitochondrial Ca2+ accumulation in spinobulbar muscular atrophy skeletal muscle
Source: Nat Commun. 2023 Feb 6;14:602. doi: 10.1038/s41467-023-36185-w (PMC9902403; doi:10.1038/s41467-023-36185-w)
Supplement: Supplementary file 1 — Supplementary Information [file 41467_2023_36185_MOESM1_ESM.pdf]

Defective excitation-contraction coupling and mitochondrial respiration precede mitochondrial Ca^{2+} accumulation in the skeletal muscle of patients with spinobulbar muscular atrophy

Supplementary information

List of supplementary material

Supplementary Tables

Supplementary Table 1. Description of control subjects and SBMA patients.

Supplementary Table 2. Primers used for quantitative real-time PCR analyses.

Supplementary Figures

Supplementary Fig. 1. Altered muscle contraction in SBMA.

Supplementary Fig. 2. Early and progressive changes in gene expression in SBMA transgenic mice.

Supplementary Fig. 3. Altered expression of genes involved in sarcomere organization and muscle contraction in presymptomatic SBMA knock-in mice.

Supplementary Fig. 4. Early altered expression of genes involved in muscle structure, contraction and metabolism in knock-in SBMA mice.

Supplementary Fig. 5. Altered expression of ECC genes in fast-twitch muscles of AR100Q mice.

Supplementary Fig. 6. Central core-like pathology and normal lactate levels in the bloodstream of AR100Q mice.

Supplementary Fig. 7. Disrupted sarcomere organization in the muscle of SBMA mice.

Supplementary Fig. 8. Disruption of myofiber organization in SBMA mice.

Supplementary Fig. 9. Amelioration of muscle pathology by surgical castration.

Supplementary Fig. 10. Amelioration of myofiber organization by surgical castration.

Supplementary Data

Supplementary Data 1. Transcriptome analysis in transgenic SBMA mice.

Supplementary Data 2. Transcriptome analysis in knock-in SBMA mice.

Supplementary Table 1. Description of control subjects and SBMA patients.

Control subjects	Muscle	AR Q length
1	quadriceps femoris	N/A
2	vastus lateralis	N/A
3	vastus lateralis	N/A
4	vastus lateralis	N/A
5	vastus lateralis	N/A
6	vastus lateralis	N/A
7	vastus lateralis	N/A
SBMA patients	Muscle	AR Q length
1	quadriceps femoris	44
2	vastus lateralis	44
3	vastus lateralis	49
4	vastus lateralis	44
5	vastus lateralis	47

Supplementary Table 2. Primers used for quantitative real-time PCR analyses.

	Forward	Reverse
Cacna1 s	CAGACACAGAGAGCTTGTATGAA	TTCACTAGGTCATGGCACTTC
Casq1	CCCTGTAGAGTTGATTGAAGGTGAACG	CCTCGTAGGCTTTGTAATGCTCTGAGTC
Casq2	CCAGCTGAAGGAGATTGTACTG	AGCAAGCTTGGCCTCTTT
Atp2a1	CCACCAACCAGATGTCAGTT	TCCCTCAGGAGCATAAGTAGAG
Atp2a2	CTTATCTTGGTAGCCAATGCAATC	TTGCCCATTTCAGGCTCATA
Pv	AGGTGAAGAAGGTGTTCCATATT	TCTCTGGCATCTGAGGAGGAA
Sln	GCTCCTCTTCAGGAAGTGAAG	TGGCCCCTCAGTATTGGTAGG
Ryr1	GCAACCGCCTTTGCTTTC	ACTGACAGAGACTGCTCCA
Mpc2	CCGACTCATGGATAAAGTGGAG	CCATTTCATTATTGGAGCCCAGA
Cpt1	CTGCACTCCTGGAAGAAGAAG	CTGTGCACTGAGGTATCTCTTC
Fkbp12	GTGATCCGAGGCTGGGAGGAAG	GGCTCCATAGGCATAGTCTGAGGAGA
Stac3	CACTCTACAGCGACCAACAGTA	CACTCTACAGCGACCAACAGTA
Junctin	CATGGTTCATGGTCATTGCATTGC	TCAAAGTCTCCATCGCCATCCG
Triadin	GCACCCACCATGACTGAGATCAC	AGCACCTTTCCAGGGGATTTAGG
b-actin	GACAGGATGCAGAAGGAGATTACTG	CTCAGGAGGAGCAATGATCTTGAT

Supplementary Figures

Supplementary Figure 1

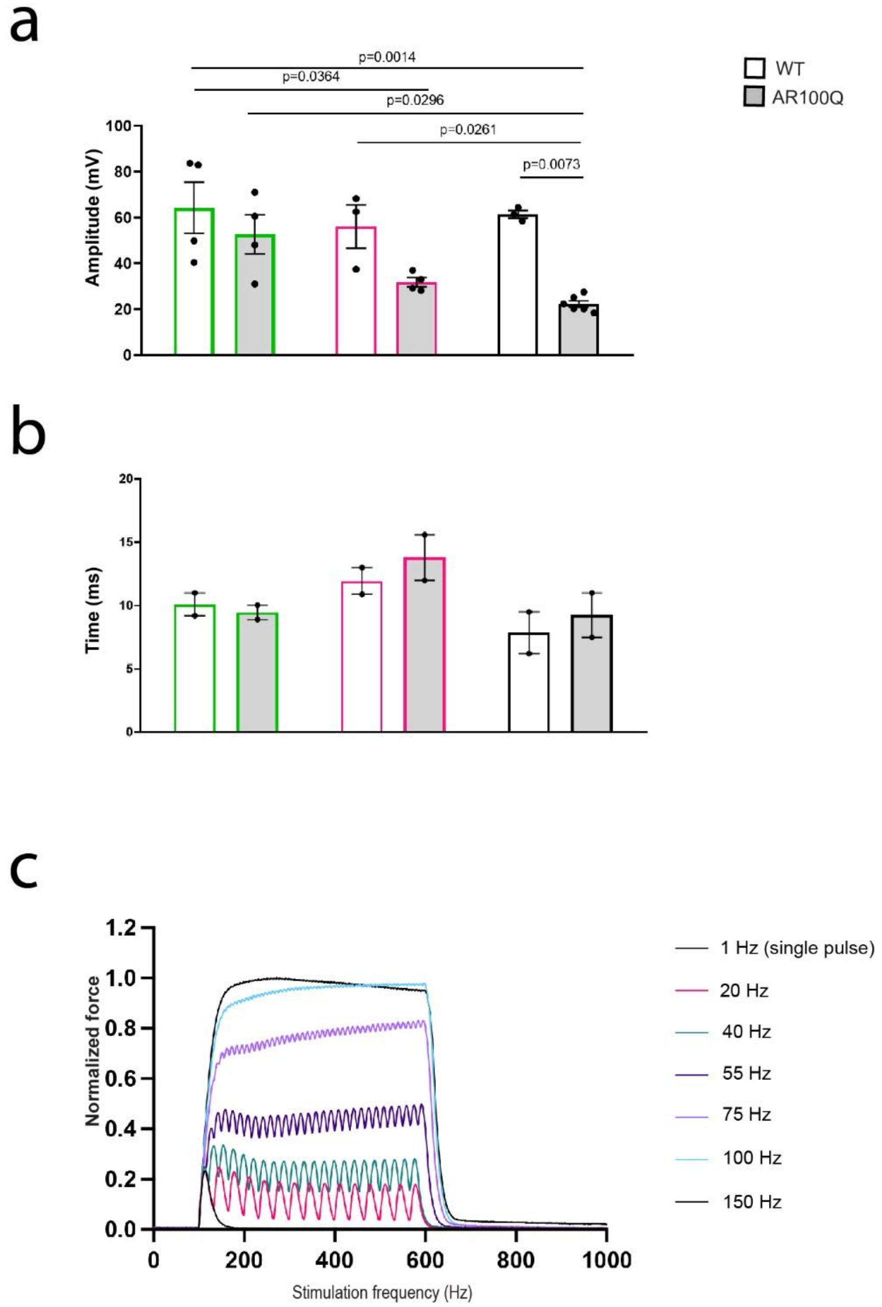

Supplementary Fig. 1. Altered muscle contraction in SBMA.

- **a)** CMAP analysis in the gastrocnemius muscle of 4-week-old (green), 8-week-old (magenta), and 12-week-old (black) WT and AR100Q mice (each dot is the average of 3 measures/muscle; n=4 mice/genotype/1M; n=3 mice/WT/2M-3M and n=6 mice/AR100Q/2M-3M).
- **b)** Analysis of the H-reflex in the *flexor digitorum brevis* muscles of 4-week-old (green), 8-week-old (magenta), and 12-week-old (black) WT and AR100Q mice (n = 2 mice/genotype/age).
- **c)** Representative traces of EDL stimulation (relative to Fig. 1c).

The graphs show the mean ± SEM; ; significance was tested with two-way ANOVA followed by Tukey's post-hoc test. Source data are provided as a Source Data file.

Supplementary Figure 2

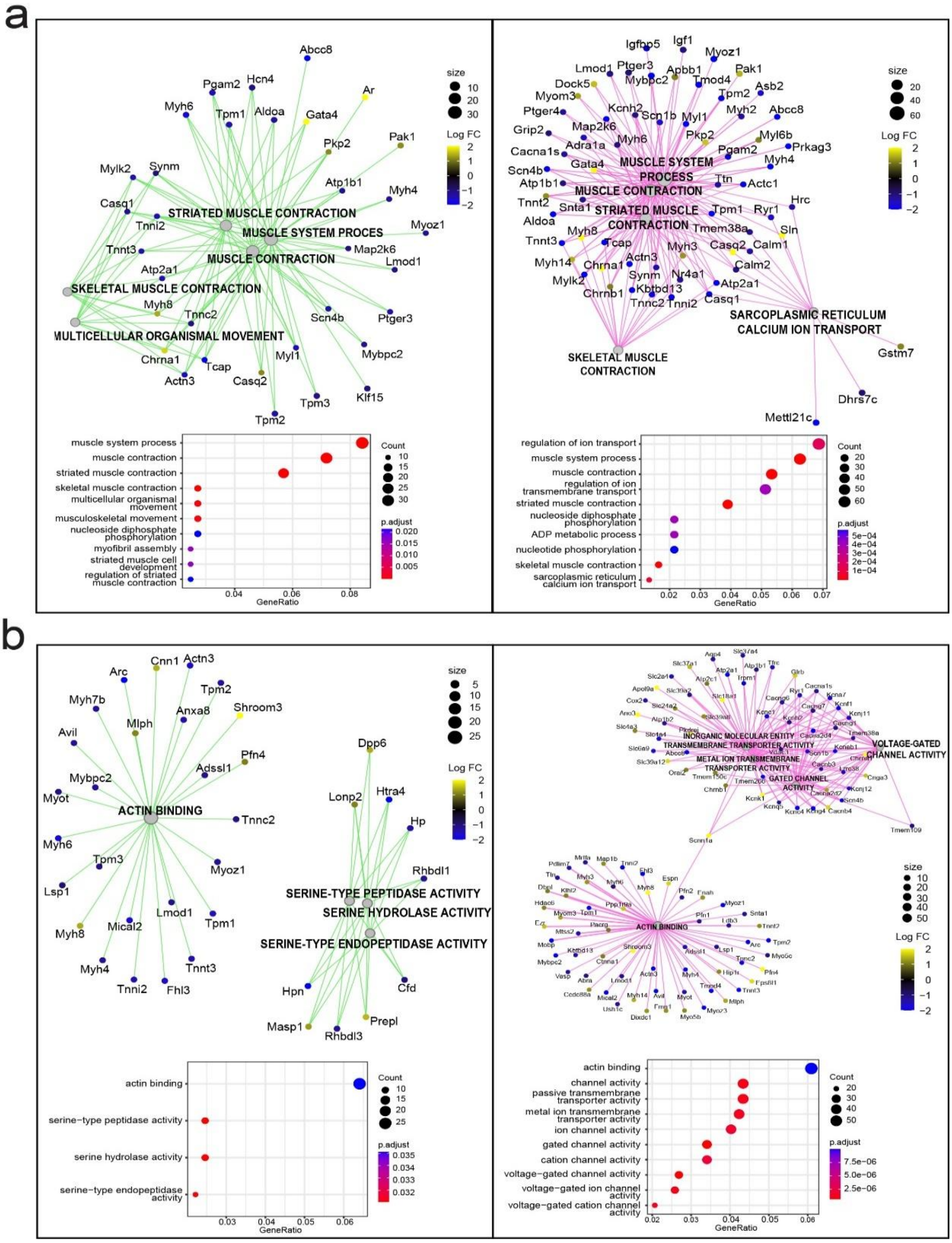

Supplementary Fig. 2. Early and progressive changes in gene expression in SBMA transgenic mice.

a) **a-b**) Transcriptomic (RNA-seq) and Gene Ontology analysis based on "biological process" (a) and "molecular function" (b) for differentially expressed genes in the quadriceps muscle of 4- and 8-week-old AR100Q mice and WT mice (n=4 mice/WT/1M; n=4 mice/AR100Q/1M; n=3 mice/WT/2M; n=4 mice/AR100Q/2M).

Supplementary Figure 3

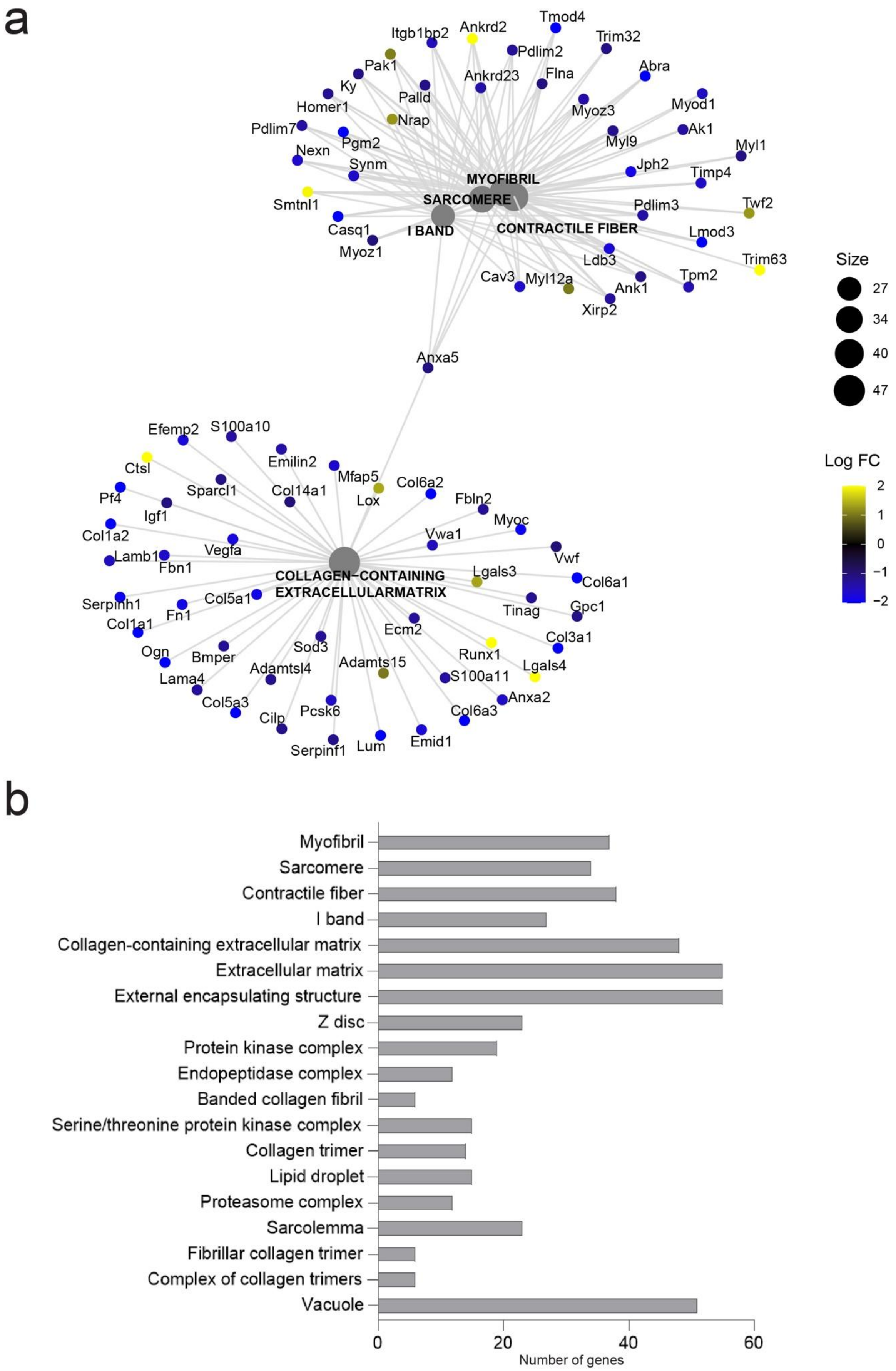

Supplementary Fig. 3. Altered expression of genes involved in sarcomere organization and muscle contraction in presymptomatic SBMA knock-in mice.

a) **a-b**) Transcriptomic (microarray) and Gene Ontology analysis based on "cellular component" for differentially expressed genes in the quadriceps muscle of 12-week-old (before onset of motor dysfunction) knock-in AR113Q mice and control mice (n=4 mice/WT, n=3 mice/AR113Q).

Supplementary Figure 4

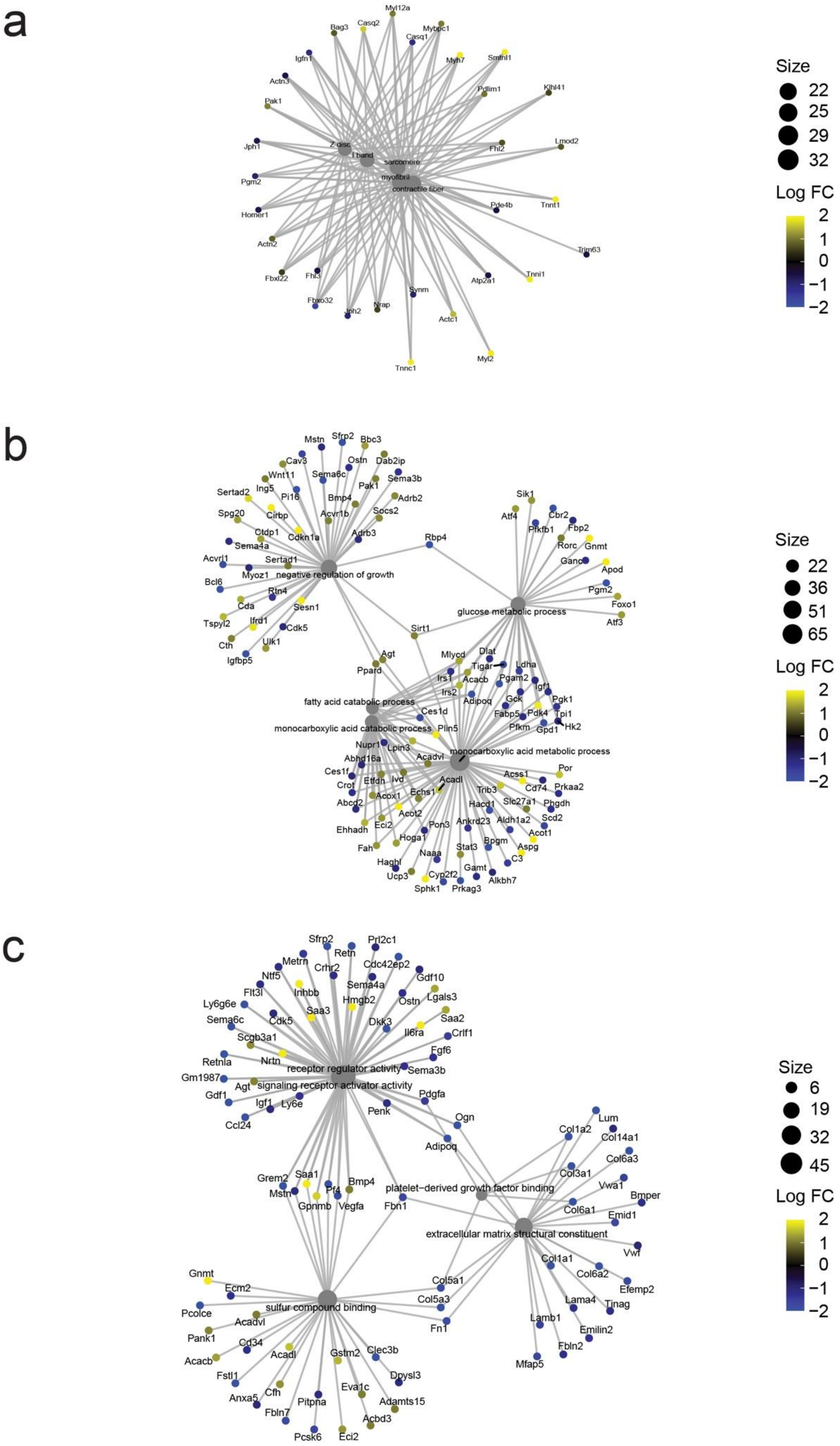

Supplementary Fig. 4. Early altered expression of genes involved in muscle structure, contraction and metabolism in knock-in SBMA mice.

- **a)** Gene Ontology analysis based on the category "cellular component" for genes that were differentially expressed between 24-week-old AR113Q and control mice (n = 3 mice/genotype/age [26]).
- **b)** Gene Ontology analysis based on the category "biological process" for genes that were differentially expressed between 12-week-old AR113Q and control mice (n=4 mice/WT, n=3 mice/AR113Q).
- **c)** Gene Ontology analysis based on the category "molecular function" for genes that were differentially expressed between 12-week-old AR113Q and control mice (n=4 mice/WT, n=3 mice/AR113Q).

Supplementary Figure 5

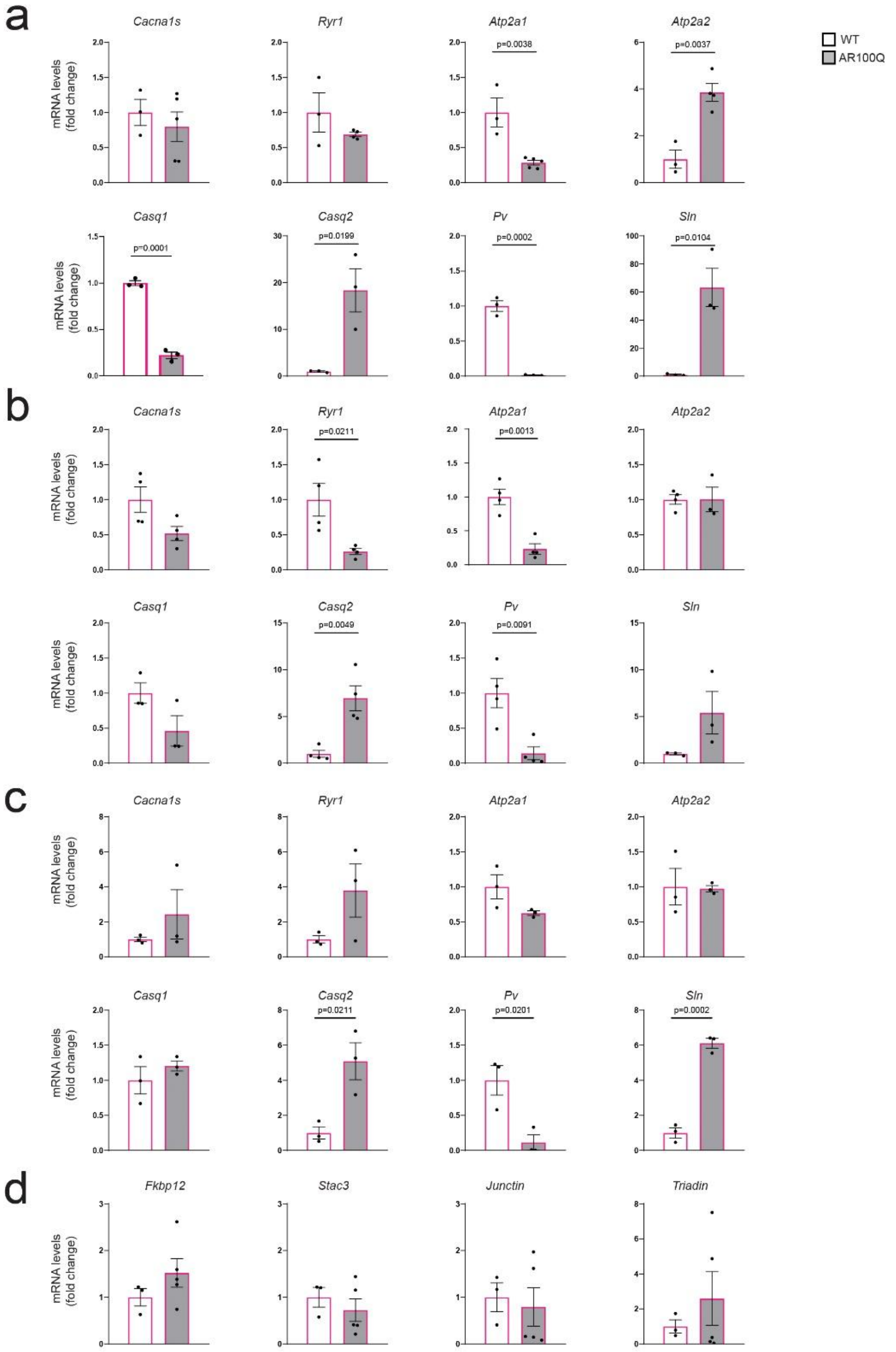

Supplementary Fig. 5. Altered expression of ECC genes in fast-twitch muscles of AR100Q mice.

a-d) RT–PCR analysis of the expression levels of the indicated genes normalized to *beta-actin* expression levels in the (a) EDL (Cacna1s, Atp2a1: n=3 mice/WT and n=5 mice/AR100Q; Ryr1, Atp2a2: n=3 mice/WT and n=4 mice/AR100Q; Casq1, Casq2, Pv, Sln: n=3 mice/WT and n=3 mice/AR100Q), (b) *flexor digitorum brevis* (Cacna1s, Ryr1, Atp2a1, Casq2, Pv: n=4 mice/WT and n=4 mice/AR100Q; Atp2a2: n=4 mice/WT and n=3 mice/AR100Q; Casq1, Sln: n=3 mice/WT and n=3 mice/AR100Q), (c) soleus (n=3 mice/genotype), and (d) quadriceps (Fkbp12: n=3 mice/WT n=4 mice/AR100Q; Stac3, Junctin, Triadin: n=3 mice/WT n=5 mice/AR100Q) muscles of 8-week-old. The graphs show the mean ± SEM, Student's t test was used for the comparisons. Source data are provided as a Source Data file.

Supplementary Figure 6

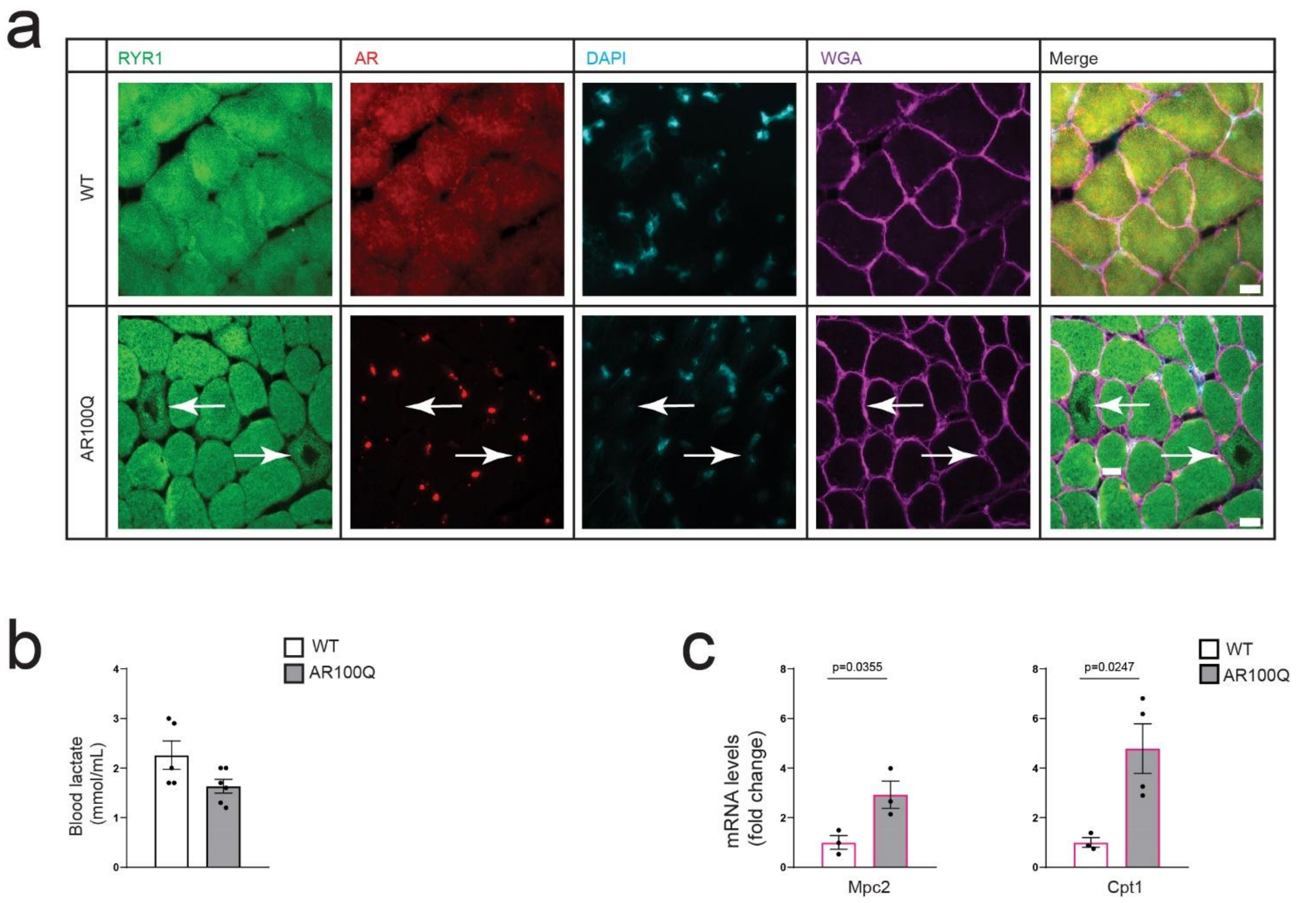

Supplementary Fig. 6. Central core-like pathology and normal lactate levels in the bloodstream of AR100Q mice.

a) Immunofluorescence analysis of RYR1 (green), AR (red), and wheat germ agglutinin (WGA, purple) expression and nuclei (DAPI, blue) in the quadriceps muscle of 8-week-old WT and AR100Q mice. Representative images of replicates obtained from 3 mice/genotype are shown. Bar, 20 µm. Arrows point to fibers with areas deprived of RYR1.

b) Analysis of blood lactate levels in 12-week-old mice (n = 5 mice/WT and n=6 mice/AR100Q).

c) RT–PCR analysis of the transcript levels of the indicated genes normalized to *beta-actin* transcript levels in the quadriceps muscle of 8-week-old WT and AR100Q mice (Mpc2: n=3 mice/genotype; Cpt1: n=3 mice/WT and n=4 mice/AR100Q).

The graphs show the mean ± SEM, Student's t test. Source data are provided as a Source Data file.

Supplementary Figure 7

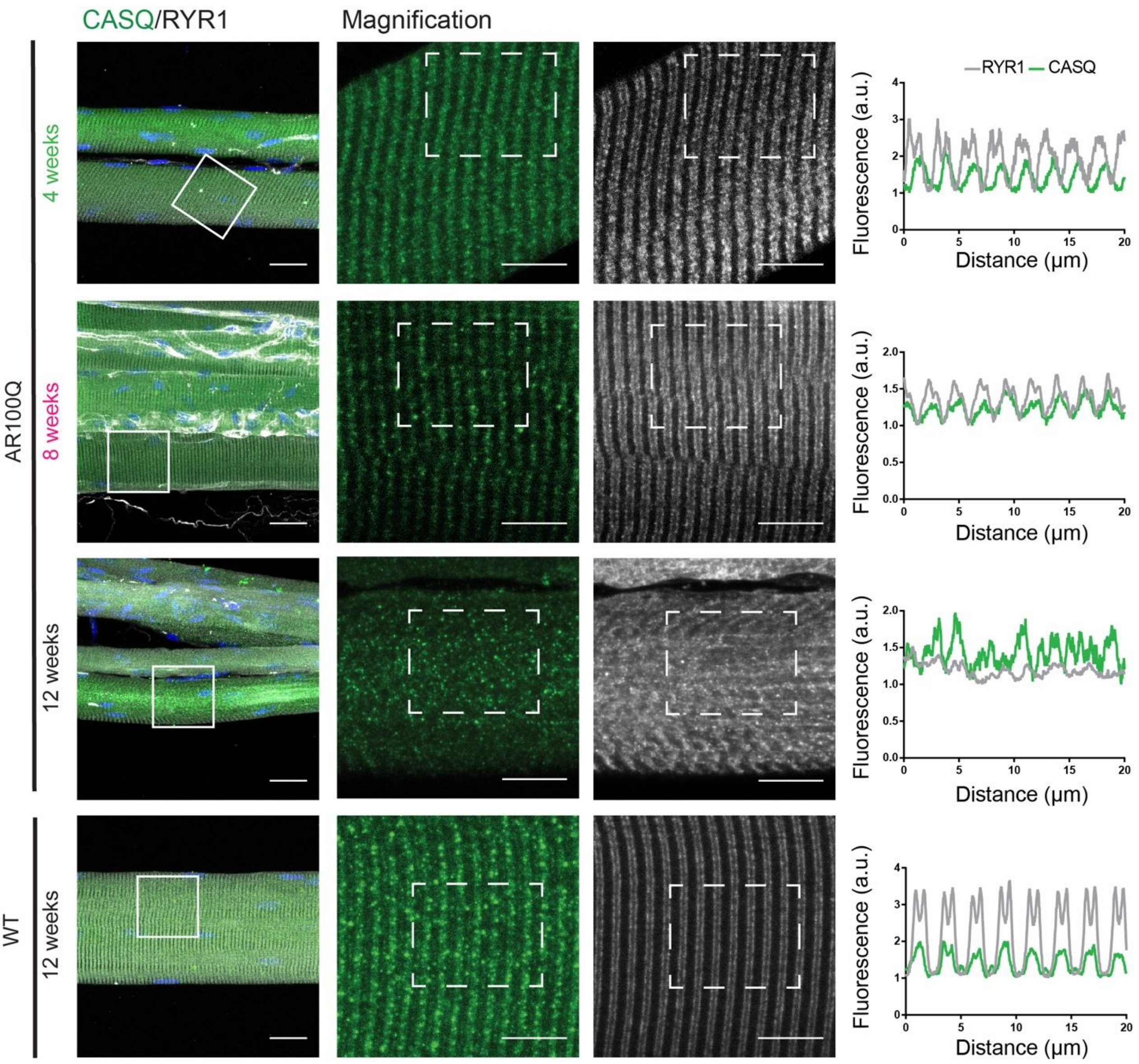

Supplementary Fig. 7. Disrupted sarcomere organization in the muscle of SBMA mice.

Immunofluorescence and confocal microscopy analysis of RYR1 (white) and CASQ (green) expression in fibers isolated from the TA muscle of WT and AR100Q mice (n = 3 mice/genotype/age). Representative images are shown. Bar = 25 µm, magnification bar = 10 µm. The graphs show fluorescence quantification (described in the Methods section).

Supplementary Figure 8

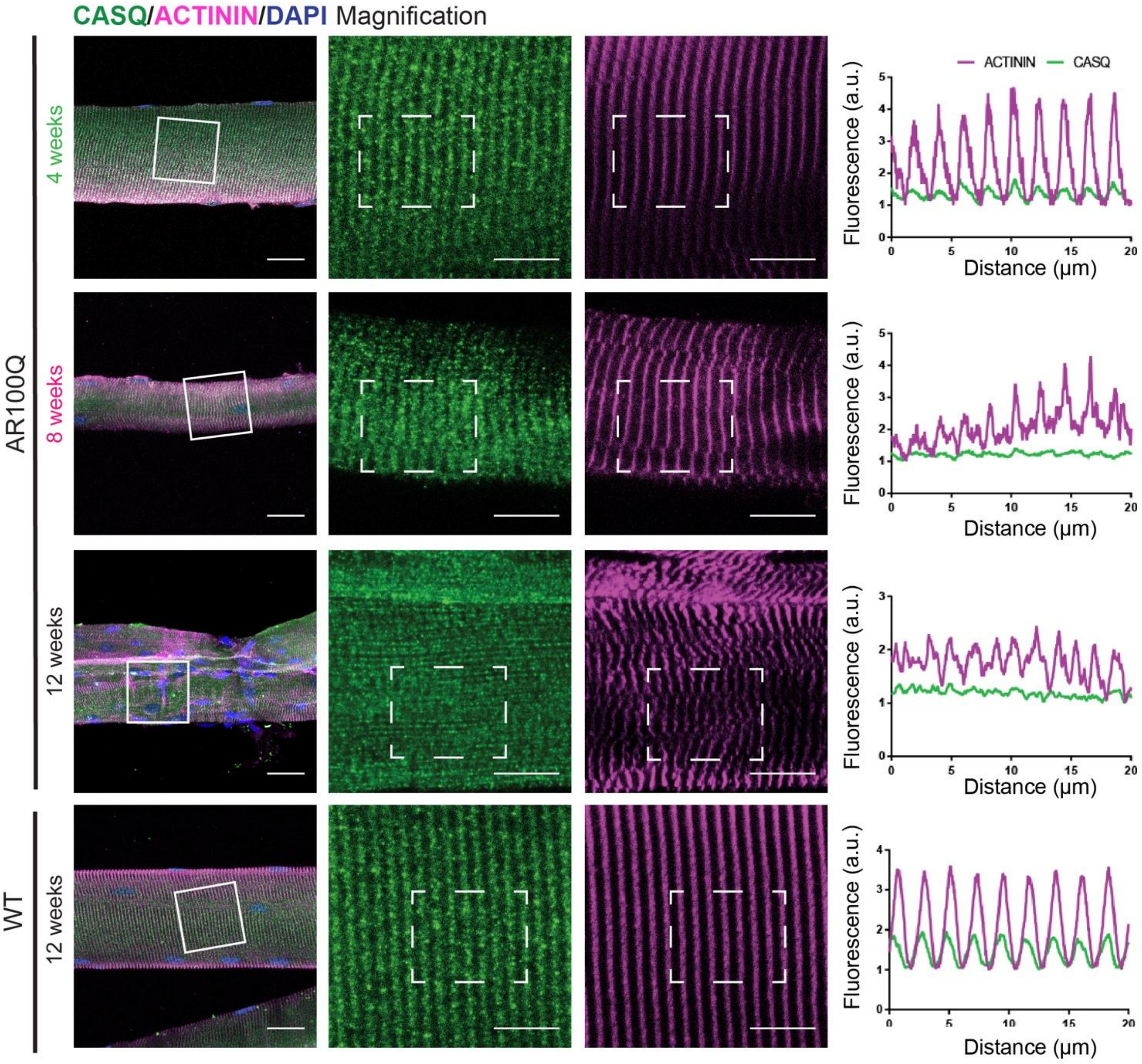

Supplementary Fig. 8. Disruption of myofiber organization in SBMA mice.

Immunofluorescence and confocal microscopy analysis of Actinin (purple) and CASQ (green) expression, and DAPI (blue) fluorescence in myofibers isolated from the TA muscle of WT and AR100Q mice (n = 3 mice/genotype/age). Representative images are shown. Bar = 25 µm, Magnification bar = 10 µm. The graphs show fluorescence quantification (described in the Methods section).

Supplementary Figure 9

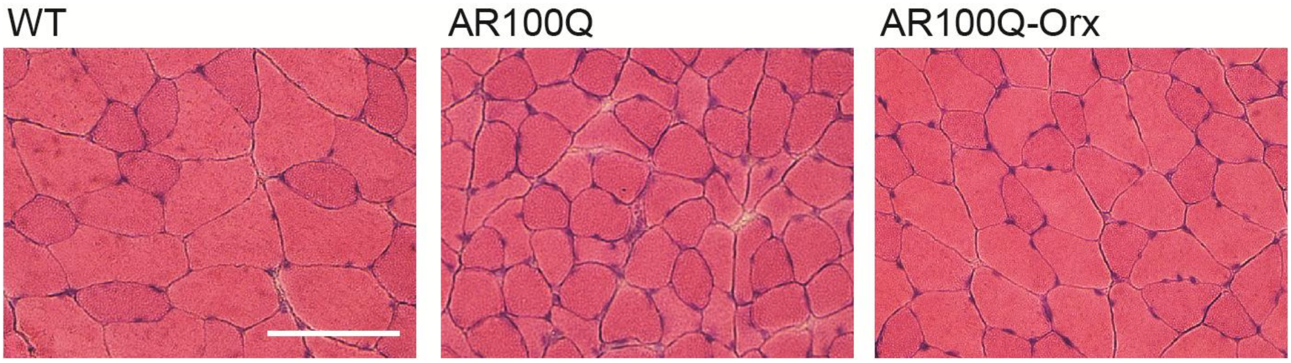

Supplementary Fig. 9. Amelioration of muscle pathology by surgical castration.
Hematoxylin and eosin analysis of the quadriceps muscle of sham-operated or orchiectomized (Orx) 8-week-old WT and AR100Q mice (n = 3 mice/genotype). Representative images are shown. Bar = 100 μm.

Supplementary Figure 10

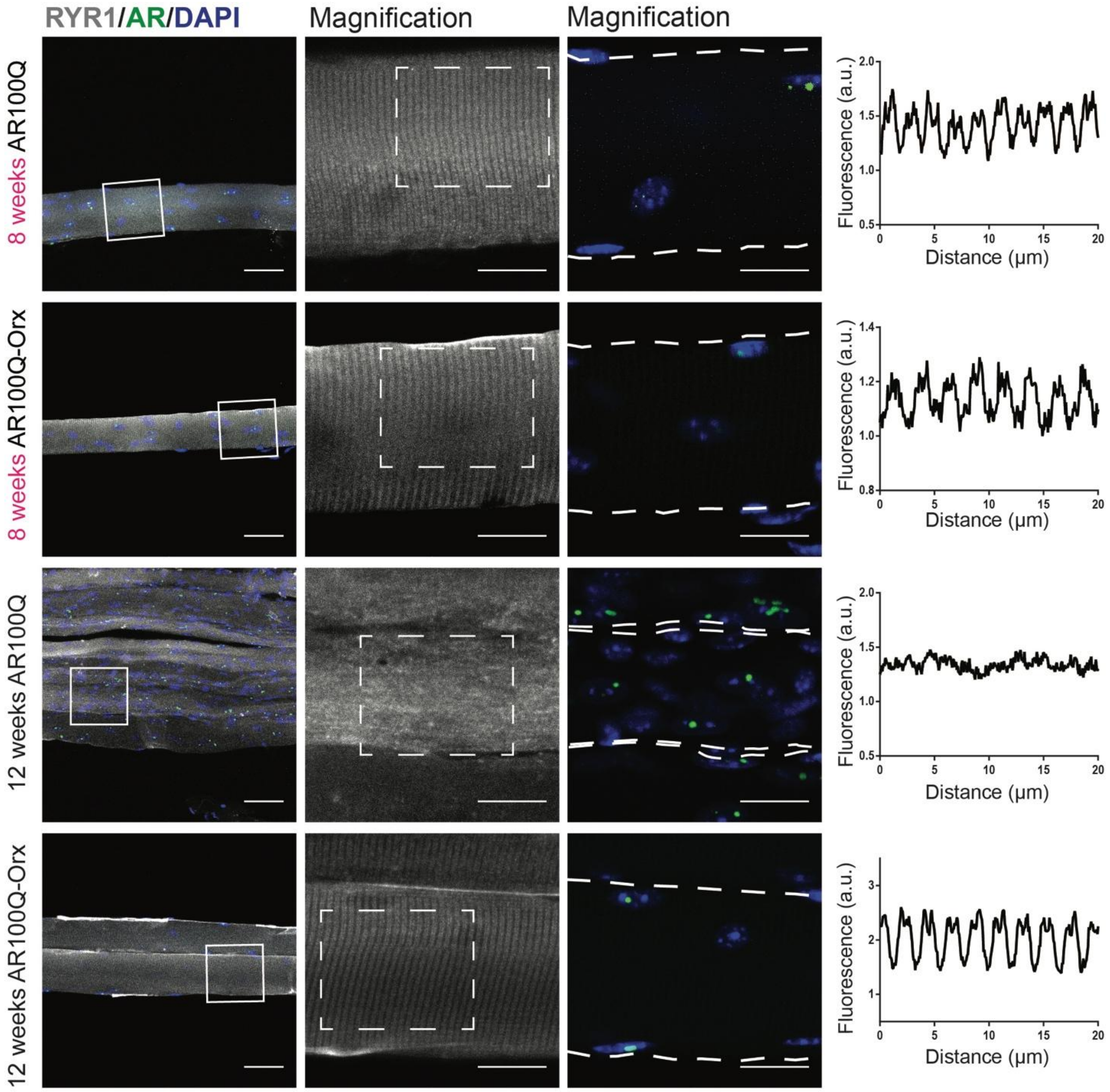

Supplementary Figure 10. Amelioration of myofiber organization by surgical castration.

Immunofluorescence analysis of RYR (gray) and AR (green) expression and DAPI (blue) fluorescence in myofibers isolated from the TA muscle of WT and AR100Q mice (n = 3 mice/genotype/age). Representative images are shown. Bar = 25 µm, Magnification bar = 10 µm. The graphs show fluorescence quantification (described in the Methods section).
