# Supplementary material for: Defective excitation-contraction coupling and mitochondrial respiration precede mitochondrial Ca2+ accumulation in spinobulbar muscular atrophy skeletal muscle
Source: Nat Commun. 2023 Feb 6;14:602. doi: 10.1038/s41467-023-36185-w (PMC9902403; doi:10.1038/s41467-023-36185-w)
Supplement: Supplementary file 3 — Description of Additional Supplementary Files [file 41467_2023_36185_MOESM3_ESM.pdf]

Description of Additional Supplementary Files

Supplementary Data 1. Differentially expressed genes and gene ontology analysis from RNAseq transcriptome analysis in WT and AR100Q SBMA transgenic mice at 1 and 2 months of age.

Supplementary Data 2. Differentially expressed genes and gene ontology analysis from microarray transcriptome analysis in WT and AR113Q SBMA knock-in mice at 3 months of age.
