# Supplementary material for: Defective excitation-contraction coupling and mitochondrial respiration precede mitochondrial Ca2+ accumulation in spinobulbar muscular atrophy skeletal muscle
Source: Nat Commun. 2023 Feb 6;14:602. doi: 10.1038/s41467-023-36185-w (PMC9902403; doi:10.1038/s41467-023-36185-w)
Supplement: Supplementary file 6 — Reporting Summary [file 41467_2023_36185_MOESM6_ESM.pdf]

nature portfolio

Corresponding author(s): Maria Pennuto
Last updated by author(s): Dec 12, 2022

Reporting Summary

Nature Portfolio wishes to improve the reproducibility of the work that we publish. This form provides structure for consistency and transparency in reporting. For further information on Nature Portfolio policies, see our Editorial Policies and the Editorial Policy Checklist.

Statistics

For all statistical analyses, confirm that the following items are present in the figure legend, table legend, main text, or Methods section.

n/a	Confirmed	
☐	☒	The exact sample size (*n*) for each experimental group/condition, given as a discrete number and unit of measurement
☐	☒	A statement on whether measurements were taken from distinct samples or whether the same sample was measured repeatedly
☐	☒	The statistical test(s) used AND whether they are one- or two-sided<br>*Only common tests should be described solely by name; describe more complex techniques in the Methods section.*
☒	☐	A description of all covariates tested
☐	☒	A description of any assumptions or corrections, such as tests of normality and adjustment for multiple comparisons
☐	☒	A full description of the statistical parameters including central tendency (e.g. means) or other basic estimates (e.g. regression coefficient) AND variation (e.g. standard deviation) or associated estimates of uncertainty (e.g. confidence intervals)
☐	☒	For null hypothesis testing, the test statistic (e.g. *F*, *t*, *r*) with confidence intervals, effect sizes, degrees of freedom and *P* value noted<br>*Give P values as exact values whenever suitable.*
☒	☐	For Bayesian analysis, information on the choice of priors and Markov chain Monte Carlo settings
☒	☐	For hierarchical and complex designs, identification of the appropriate level for tests and full reporting of outcomes
☒	☐	Estimates of effect sizes (e.g. Cohen's *d*, Pearson's *r*), indicating how they were calculated

Our web collection on statistics for biologists contains articles on many of the points above.

Software and code

Policy information about availability of computer code

Data collection	For immunofluorescence analysis digital images were captured with....<br>For WB analysis, Alliance Q9 Mini chemidoc system (Uvitec, Cambridge, UK) and Chemidoc (Bio-Rad) were used.
Data analysis	Statistical analysis was performed with Jamovi (v2.3.18.0) or Microsoft Office Excell (Microsoft, v2017). RNAseq data analysis was performed using Rosalind framework, cutadapt tool (v3.4), FastQC tool (v0.11.9), STAR aligner (v2.5.2b), HTSeq-count tool (v0.5.4p3) and DEseq2 tool (v1.24.0). QuantStudio™ 5 Real-Time PCR System (Thermo Fisher) was used for setting up and collecting data for RT-PCR. BioRender was used for drawing figures.<br>Panel figures were prepared with Adobe Illustrator (v.27.0.1).

For manuscripts utilizing custom algorithms or software that are central to the research but not yet described in published literature, software must be made available to editors and reviewers. We strongly encourage code deposition in a community repository (e.g. GitHub). See the Nature Portfolio guidelines for submitting code & software for further information.

Data

Policy information about availability of data

All manuscripts must include a data availability statement. This statement should provide the following information, where applicable:

- Accession codes, unique identifiers, or web links for publicly available datasets
- A description of any restrictions on data availability
- For clinical datasets or third party data, please ensure that the statement adheres to our policy

All data generated or analyzed during this study are included in this article and its Supplementary Information files. All requests for raw data and materials should be addressed to the corresponding author. Any data and materials that can be shared will be released via a material transfer agreement. Source data are provided with this paper.

Human research participants

Policy information about studies involving human research participants and Sex and Gender in Research.

Reporting on sex and gender	Information provided: Site of biopsy and CAG repeat length
Population characteristics	This information is reported in Supplementary Table 1
Recruitment	Deanonymized control and patient biopsy samples were obtained from the Neuromuscular Bank of Tissues and DNA Samples, Telethon Network of Genetic Biobanks, and of the EuroBioBank Network, and Orthopedics and Orthopedic Oncology (University-Hospital of Padua.
Ethics oversight	The protocol for muscle biopsies collection was approved by the Ethical committee for Clinical Practice of the Azienda Ospedale Università of Padova.

Note that full information on the approval of the study protocol must also be provided in the manuscript.

Field-specific reporting

Please select the one below that is the best fit for your research. If you are not sure, read the appropriate sections before making your selection.

☒ Life sciences ☐ Behavioural & social sciences ☐ Ecological, evolutionary & environmental sciences

For a reference copy of the document with all sections, see nature.com/documents/nr-reporting-summary-flat.pdf

Life sciences study design

All studies must disclose on these points even when the disclosure is negative.

Sample size	For studies involving mice, the sample size/group was determined based on the results and the variability of the outcome measures described in Chivet et al., 2020; Rocchi et al., 2016; Milioto et al., 2017, with similar methodologies or mouse models. The number of independent experiments and cells analyzed/experiment was determined based on the results and variability of the outcome measures in previous publications performing similar assays, as shown in Scaramuzzino et al., 2015; Milioto et al., 2017; Chivet et al., 2020; Rocchi et al., 2016; Palazzolo et al., 2007, 2009, 2010.
Data exclusions	No data were excluded from the analyses.
Replication	All replicates are biological replicates. The numerosity is indicated in the caption of each Figure.
Randomization	Mice were randomized and assignes to the different experimental groups.
Blinding	In each experiment, including the in vivo preclinical study, the operator was blind for genotype and treatment.

Reporting for specific materials, systems and methods

We require information from authors about some types of materials, experimental systems and methods used in many studies. Here, indicate whether each material, system or method listed is relevant to your study. If you are not sure if a list item applies to your research, read the appropriate section before selecting a response.

Materials & experimental systems

n/a	Involved in the study	
☐	☒	Antibodies
☐	☒	Eukaryotic cell lines
☒	☐	Palaeontology and archaeology
☐	☒	Animals and other organisms
☒	☐	Clinical data
☒	☐	Dual use research of concern

Methods

n/a	Involved in the study	
☒	☐	ChIP-seq
☒	☐	Flow cytometry
☒	☐	MRI-based neuroimaging

Antibodies

Antibodies used

For immunofluorescence analysis: BF-F3 (MyHC-IIb; 1:300), AR (H280, 1:200), RYR1 (MA3-925, 1:200), and WGA (1:500, W11261). For

Western Blot analysis: calnexin (ADI-SPA-860, 1:5000), RYR1 (#MA3-925, 1:2000), CASQ (VIIID12; 1:3000), SERCA1 (VE121G9, 1:50000), SERCA2 (N19; 1:1000), PV (ab11427, 1:50000), and SLN (ref. 54).
Goat anti-rabbit HRP Biorad 1706515: https://www.bio-rad.com/it-it/sku/1706515-goat-anti-rabbit-igg-h-l-hrp-conjugate?ID=1706515
Goat anti-mouse HRP Biorad 1706516: https://www.bio-rad.com/it-it/sku/1706516-goat-anti-mouse-igg-h-l-hrp-conjugate?ID=1706516
All the information related to the antibodies used in this paper are listed also in the Methods section.

Validation

IFs were always run in parallel with negative controls (not incubated with primary antibody).
The validation of the commercial antibodies is available on manufacturer's websites.
All antibodies were validated in control (wild-type mice or mock conditions), as well as by western blot or by Immunofluorescence, according to manufacturers' instructions and recommendations.For RT-PCR experiments, each pair of primers was tested with a standard curve with serial dilutions performed for each cell line and tissue analyzed here.

Eukaryotic cell lines

Policy information about cell lines and Sex and Gender in Research

Cell line source(s)

N/A

Authentication

Describe the authentication procedures for each cell line used OR declare that none of the cell lines used were authenticated.

Mycoplasma contamination

Confirm that all cell lines tested negative for mycoplasma contamination OR describe the results of the testing for mycoplasma contamination OR declare that the cell lines were not tested for mycoplasma contamination.

Commonly misidentified lines
(See ICLAC register)

Name any commonly misidentified cell lines used in the study and provide a rationale for their use.

Animals and other research organisms

Policy information about studies involving animals; ARRIVE guidelines recommended for reporting animal research, and Sex and Gender in Research

Laboratory animals

Mus musculus. The mice were pathogen free according to the FELASA list (FELASA 2014). Animals were housed in a single ventilated cage (Tecniplast Green Line Sealsafe PLUS Mouse) with autoclaved commercial soil bedding, food and enrichment. Mice were fed with a certified rodent diet (SDS VRF1 (P)). Background C57Bl6/J mice.

Wild animals

No wild animals were used in the study.

Reporting on sex

We specified that we used male mice.

Field-collected samples

Field-collected samples are not involved in this study.

Ethics oversight

Animal care protocols conform with the appropriate national legislation (art. 31, D.lgs. 26/2014) and guidelines of the Council of the European Communities (2010/63/UE) and were approved by local ethics committees (Universities of Trento with protocol number 3FAF3.49 and approval number 974/2020-PR, and Padova, Italy, approval numbers 1289/2019-PR, 207/2020-PR) and the Italian Ministry of Health.

Note that full information on the approval of the study protocol must also be provided in the manuscript.
