# Supplementary material for: Defective excitation-contraction coupling and mitochondrial respiration precede mitochondrial Ca2+ accumulation in spinobulbar muscular atrophy skeletal muscle
Source: Nat Commun. 2023 Feb 6;14:602. doi: 10.1038/s41467-023-36185-w (PMC9902403; doi:10.1038/s41467-023-36185-w)
Supplement: Supplementary file 7 — Source Data [file 41467_2023_36185_MOESM7_ESM.zip › uncropped file.pdf]

Mouse

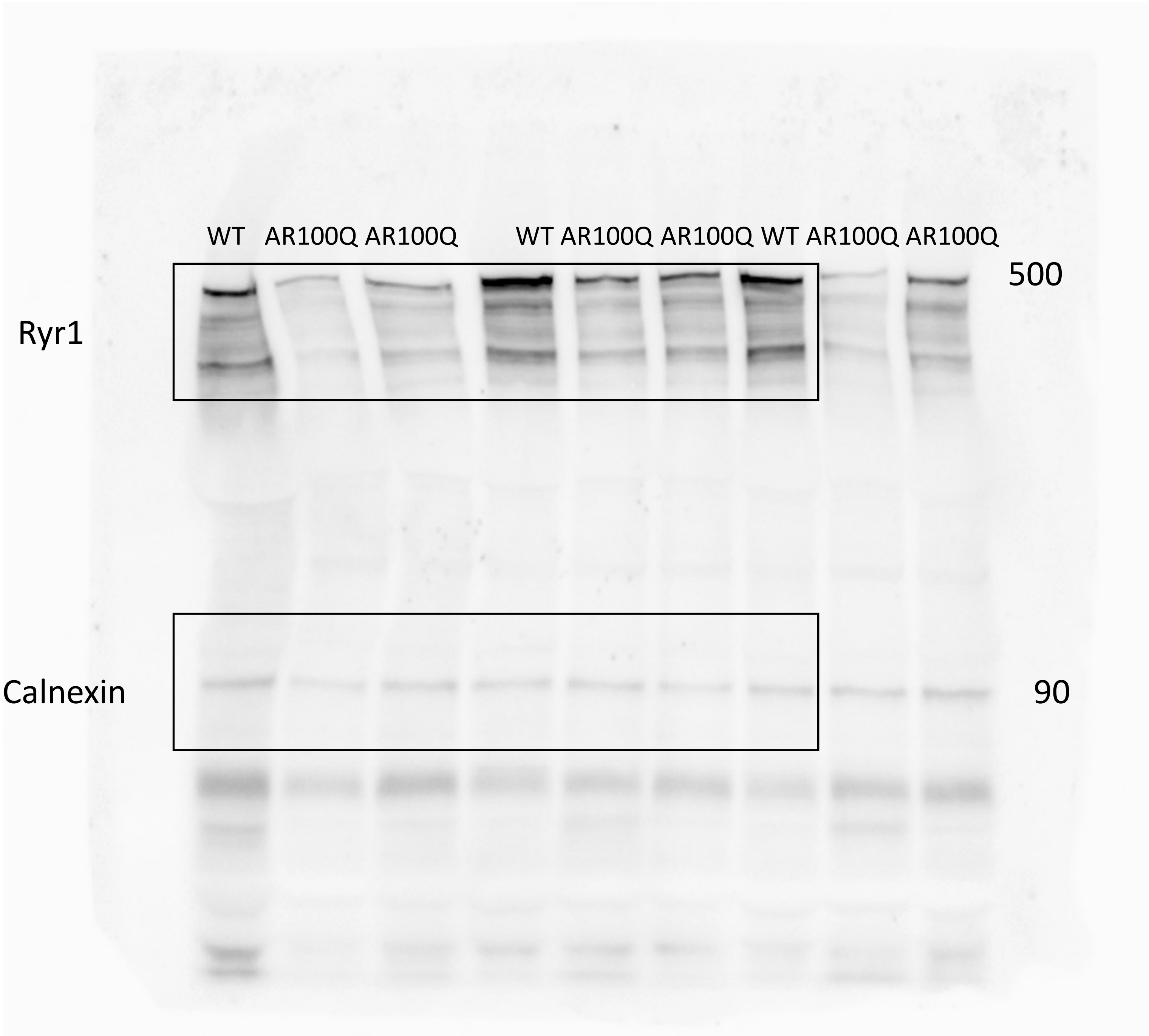

WT AR100Q AR100Q WT AR100Q AR100Q WT AR100Q AR100Q
Ryr1
500
Calnexin
90

Mouse

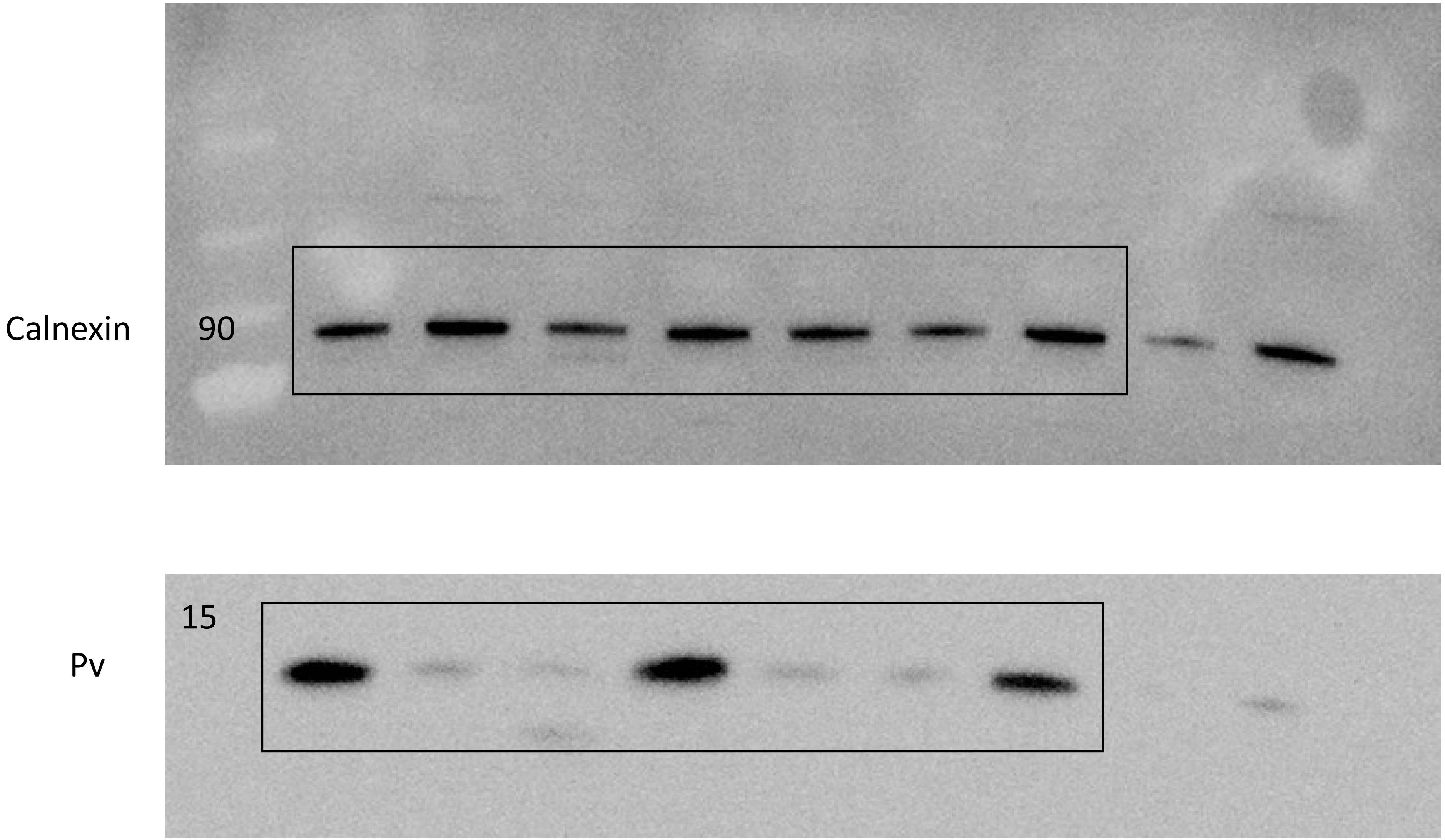

Mouse

Ponceau

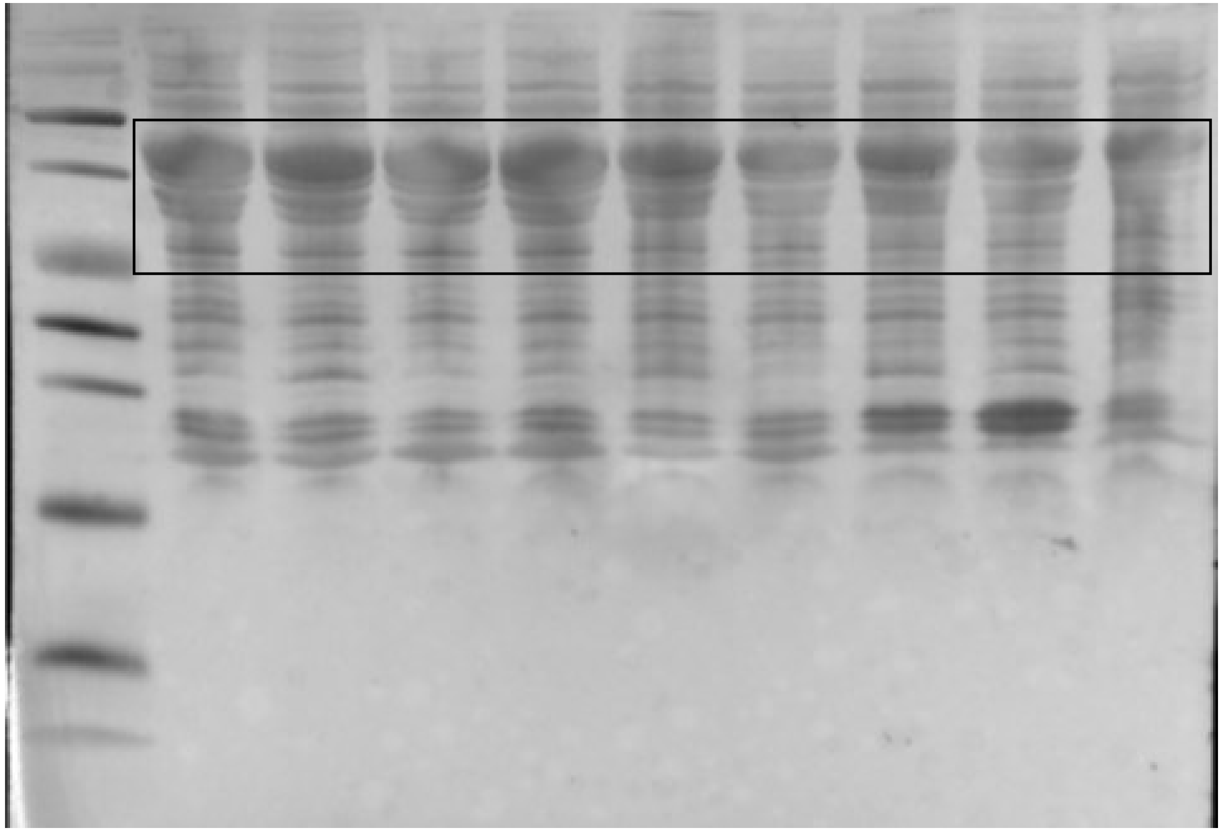

Sln

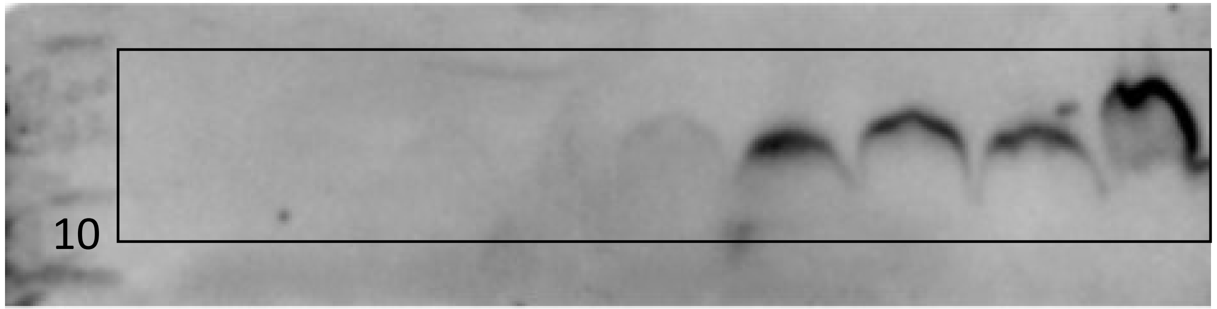

Mouse

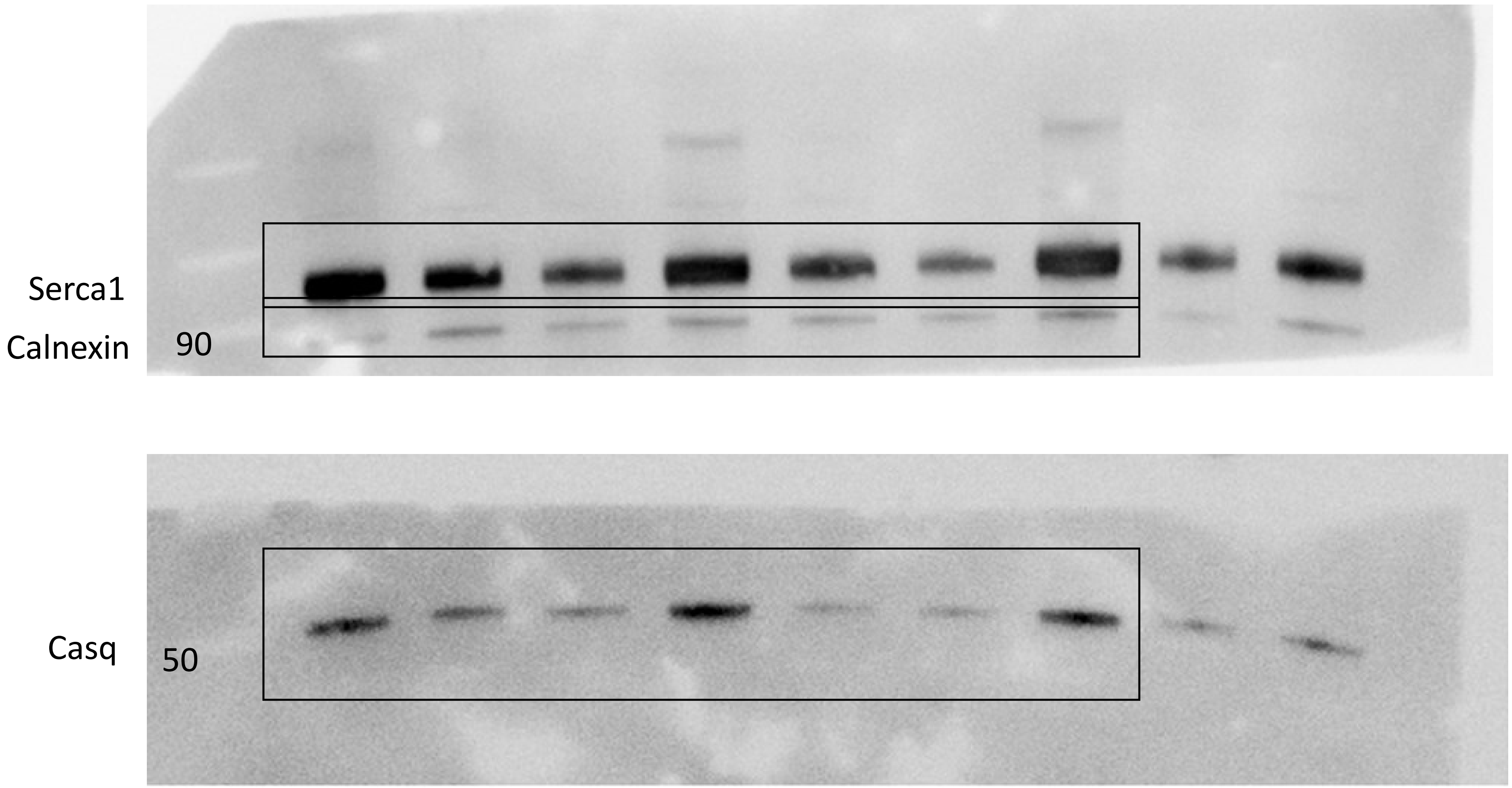

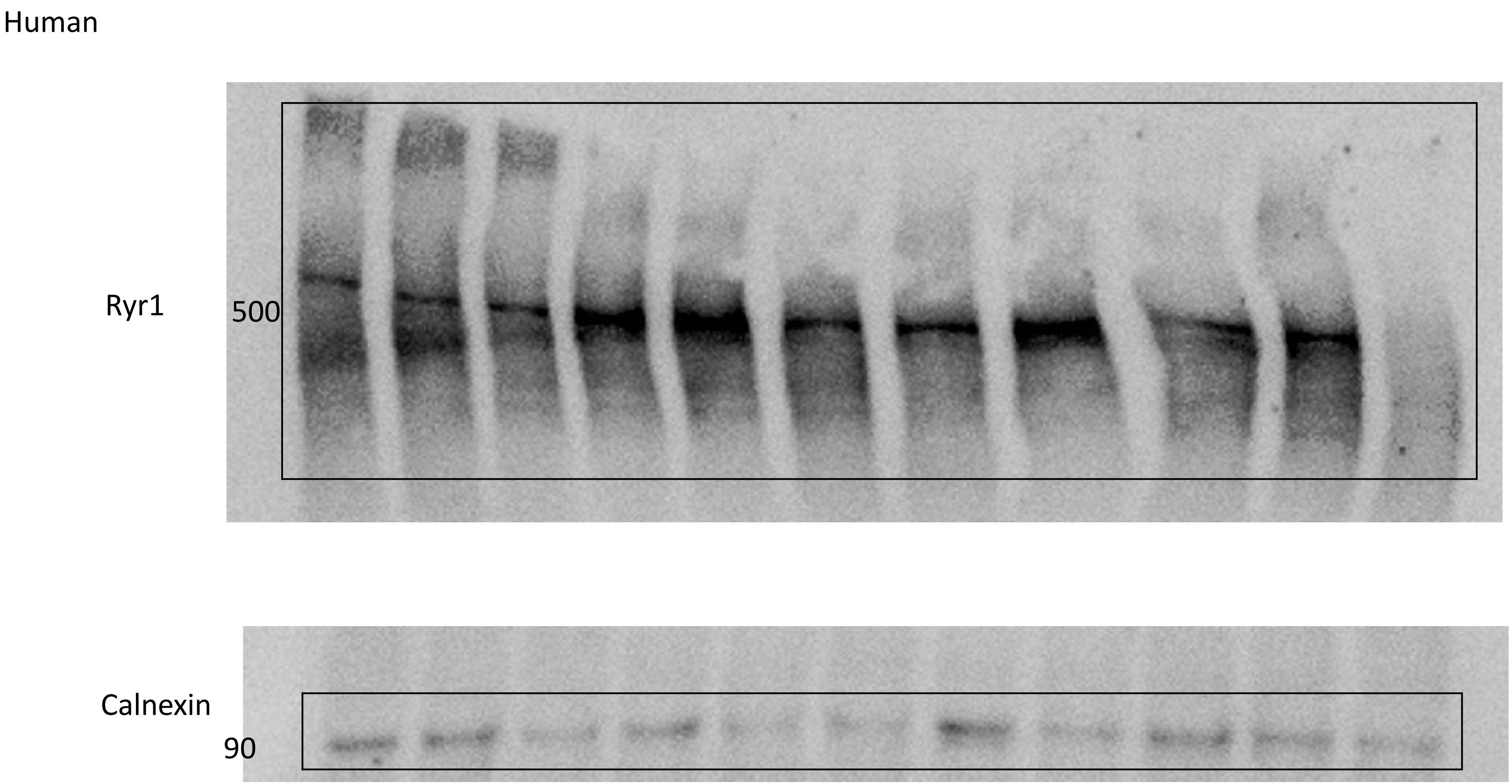
Human
Ryr1
500
Calnexin
90

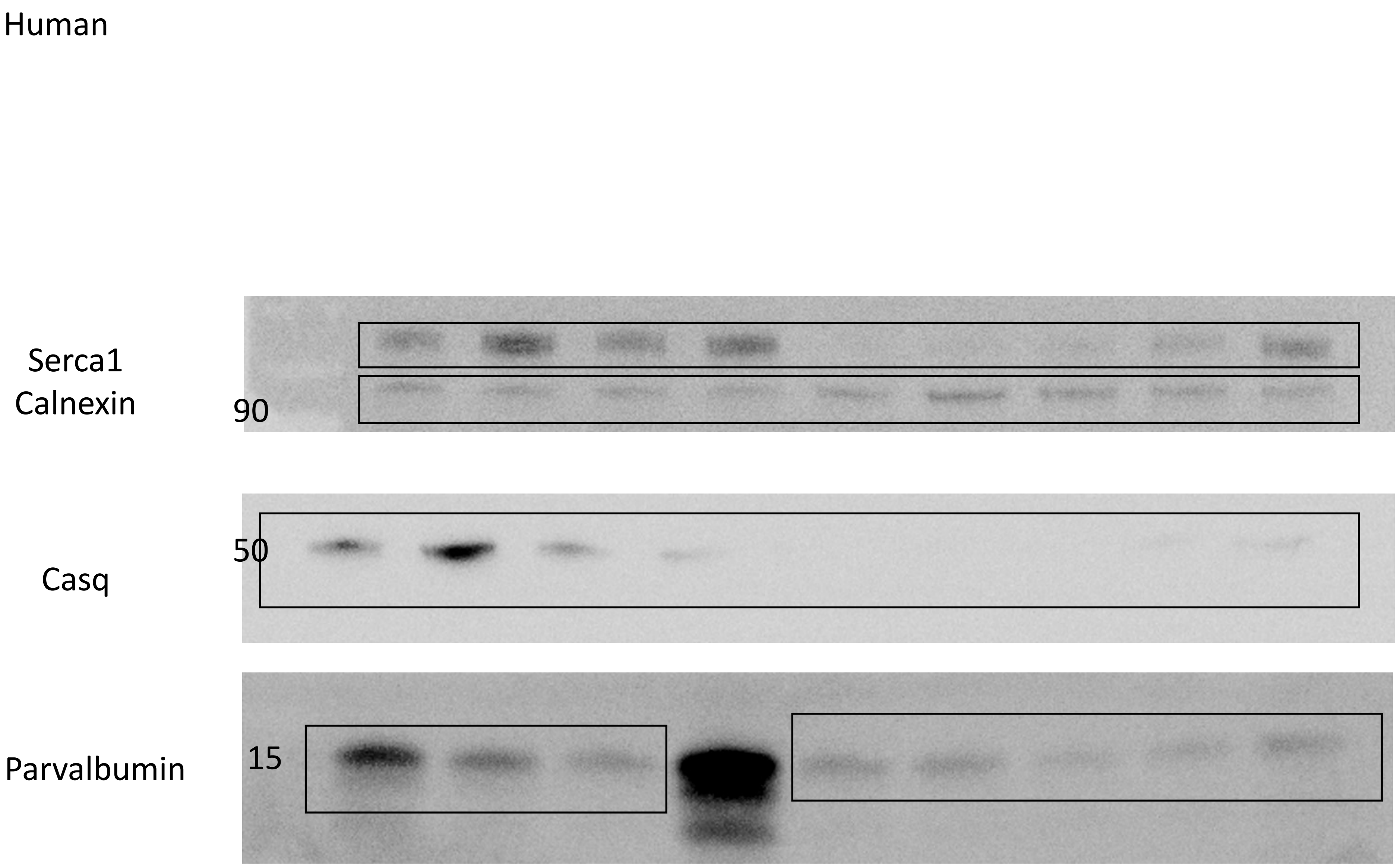
Human
Serca1
Calnexin
90
50
Casq
15
Parvalbumin

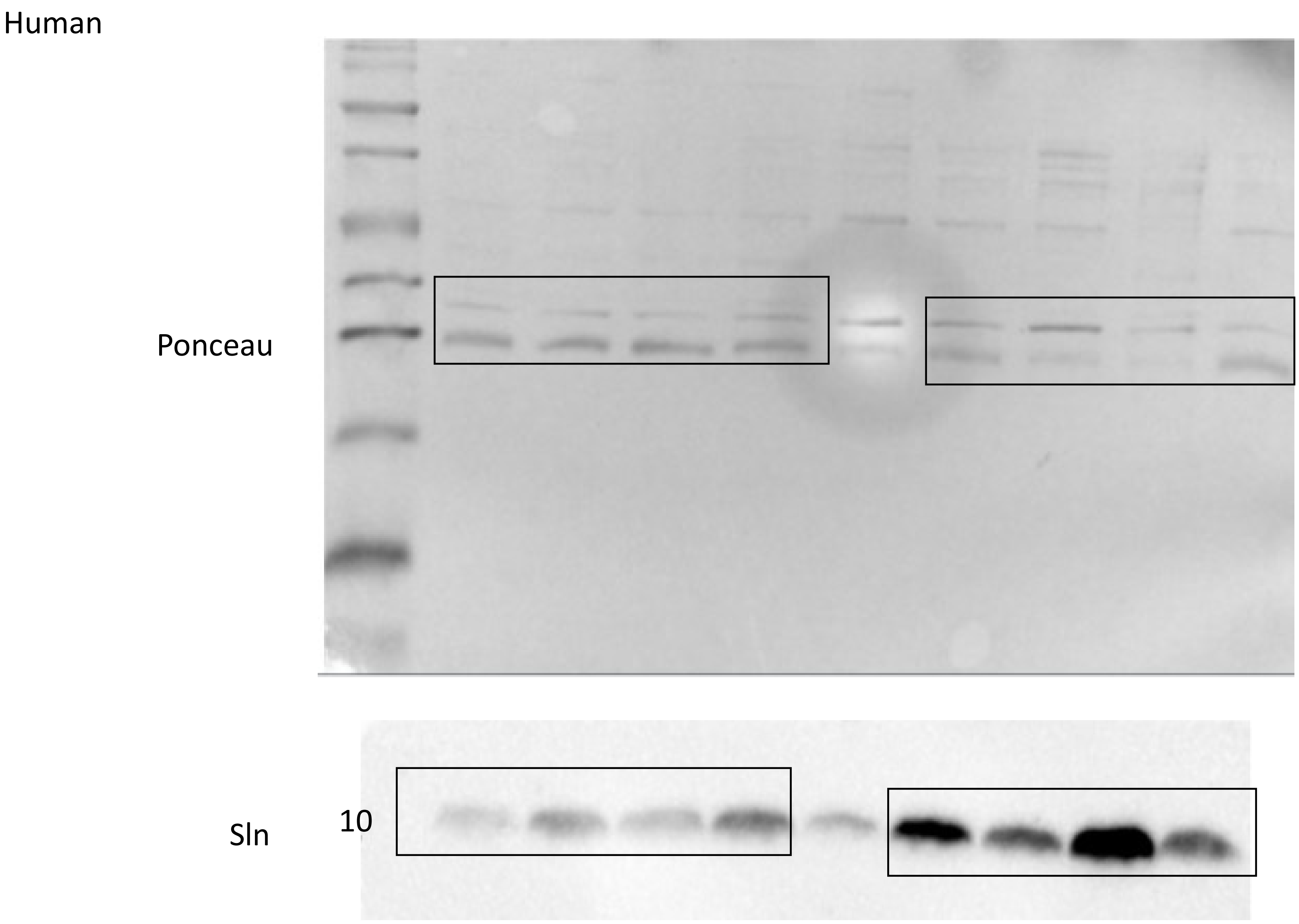
Human
Ponceau
Sln
10
